# Sex and age differences in the proportion of experienced symptoms by SARS-CoV-2 serostatus in a community-based cross-sectional study

**DOI:** 10.1017/S0950268822001339

**Published:** 2022-08-10

**Authors:** Demi M. E. Pagen, Stephanie Brinkhues, Nicole H. T. M. Dukers-Muijrers, Casper D. J. den Heijer, Noortje Bouwmeester-Vincken, Daniëlle A. T. Hanssen, Linda M. van de Laar, Inge H. M. van Loo, Paul H. M. Savelkoul, Christian J. P. A. Hoebe

**Affiliations:** 1 Department of Sexual Health, Infectious Diseases, and Environmental Health, South Limburg Public Health Service, Heerlen, The Netherlands; 2 Department of Social Medicine, Care and Public Health Research Institute (CAPHRI), Maastricht University, Maastricht, The Netherlands; 3 Department of Health Promotion, Care and Public Health Research Institute (CAPHRI), Maastricht University, Maastricht, The Netherlands; 4 Department of Infectious Diseases, North Limburg Public Health Service, Venlo, The Netherlands; 5 Department of Medical Microbiology, Maastricht University Medical Centre (MUMC+), Care and Public Health Research Institute (CAPHRI), Maastricht, The Netherlands; 6 Department of Medical Microbiology and Infection Control, Amsterdam Medical Centre, location VUmc, Amsterdam, The Netherlands

**Keywords:** COVID-19, SARS-CoV-2, sex difference, symptoms

## Abstract

We examined the possible sex and age differences in the proportion of experienced Coronavirus Disease 2019 (COVID-19) symptoms in unaware (previously) infected adults, and their uninfected counterparts, estimated by serostatus prior to vaccination, at the end of 2020 (Wuhan strain). A cross-sectional community-based study using a convenience sample of 10 001 adult inhabitants of a southern Dutch province, heavily affected by COVID-19, was conducted. Participants donated a blood sample to indicate past infection by serostatus (positive/negative). Experienced symptoms were assessed by questionnaire, before the availability of the serological test result. Only participants without confirmed SARS-CoV-2 infection were included (*n* = 9715, age range 18–90 years). The seroprevalence was comparable between men (17.3%) and women (18.0%), and participants aged 18–60 years (17.3%) and aged 60 years and older (18.6%). We showed sex and age differences in the proportion experienced symptoms by serostatus in a large cohort of both unaware (untested) seropositive compared with seronegative reference participants. Irritability only differed by serostatus in men (independent of age), while stomach ache, nausea and dizziness only differed by serostatus in women aged 60 years and older. Besides, the proportion of experiencing pain when breathing and headache differed by serostatus in men aged 18–60 years only. Our study highlights the importance of taking possible sex and age differences into account with respect to acute and long-term COVID-19 outcomes. Identifying symptom profiles for sex and age subgroups can contribute to timely identification of infection, gaining importance once governments currently move away from mass testing again.

## Introduction

The current pandemic caused by the Severe Acute Respiratory Syndrome Coronavirus 2 (SARS-CoV-2), resulting in Coronavirus Disease 2019 (COVID-19), still poses a great challenge. Worldwide, by March 2022, 445 million confirmed COVID-19 cases are counted, accompanied by almost 6 million deaths [1]. Newly emerging variants of the virus facilitate new waves of infection, despite extensive infection prevention measures taken since the start of the pandemic.

Sex and age differences may be present in the specific symptoms experienced during SARS-CoV-2 infection, although data is scarce. Among hospitalised (deceased) patients and residents of a nursing home, coughing, chills, fever and shortness of breath were reported more often in men [2, 3], while nausea was more common in women [4]. Headache and loss of smell were more often reported in COVID-19 confirmed women with mild to moderate symptoms, while fever and breathlessness were more frequent in men [5]. Further, COVID-19 confirmed women were four to five times more likely to experience cough and loss of smell or taste compared to men, whereas no significant sex differences in experienced symptoms were demonstrated in suspected COVID-19 cases [6]. Regarding age, an Estonian seroprevalence study revealed significant associations between fever, diarrhoea, and the absence of runny nose and cough with seropositivity in participants aged 50 years and older [7]. The study populations of previous research often include only confirmed (tested) and severely ill COVID-19 cases. They may represent a selected population with specific symptoms (indicating testing), thereby limiting generalisability to the overall population, including untested individuals. To obtain more information on this issue, data on symptoms in unaware infected men and women and their negative counterparts is needed.

The variety of established COVID-19-related symptoms in acute infection is wide, including common cold symptoms, coughing, shortness of breath, fever or sudden loss of smell or taste [8–10]. The variety and nature of these, sometimes unspecific and relatively mild, symptoms make it difficult to distinguish COVID-19 symptoms from symptoms caused by other flu or respiratory-like viruses in the absence of testing. Identification of experienced symptoms in specific sex and age subgroups can contribute to the timely identification of infection. In the first pandemic wave, the first half of 2020 (Wuhan strain), diagnostic testing to detect infection and interrupt further transmission timely was restricted in many countries. Though, testing was applied broader since then. The lack of testing during the first months of the pandemic resulted in many unaware infected men and women.

Since the end of 2020, sensitive and specific serological tests are available, to detect SARS-CoV-2 antibodies until 7 months after infection in 92% of the cases [11]. Hence, our study in a large cohort of unaware (previously) infected men and women, and their uninfected counterparts, aimed to examine possible sex and age differences in the proportion of experienced symptoms with untested (unaware) past SARS-CoV-2 infection, estimated by serostatus prior vaccination at the end of 2020.

## Method

### Study design

A cross-sectional community-based study was conducted at the end of 2020, before COVID-19 vaccines were given in the Netherlands [12]. A convenience sample of 10 001 adult inhabitants of a southern province of the Netherlands, heavily affected by COVID-19, was used. Past infection was indicated by serostatus of the participants. An extensive online questionnaire was taken before the serological test result was available.

### Participants

Participants were recruited by local open media channels and all adults (18+ years) living in the southern Dutch region were eligible for participation until the requested number of participants was reached. A reserve list was held to ensure sufficient participants after the initial 10 000 registrations (automatically allocated). Reserves were invited on first-come-first-serve base. Participants needed to understand, write and read the Dutch language, since materials were only available in Dutch.

### Study population

**Fig. 1.**
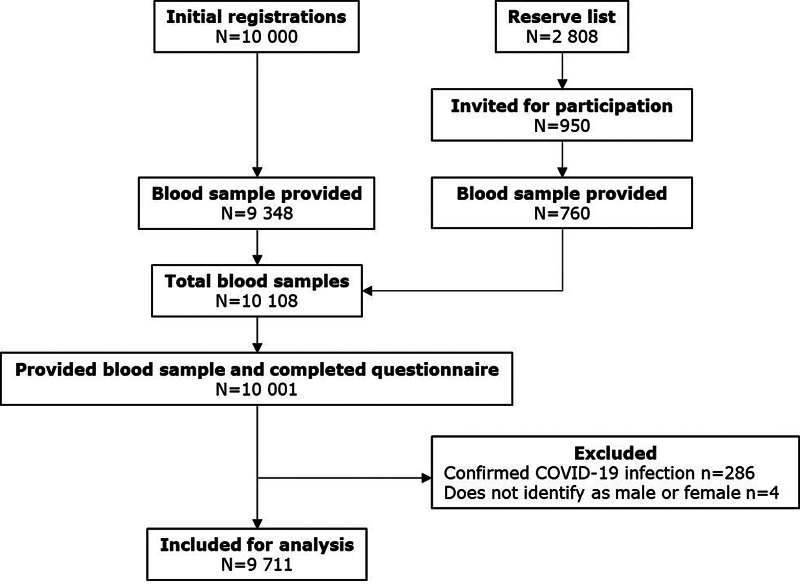
Flow chart of study population after exclusion of participants being aware of past SARS-CoV-2 infection and participants not identifying themselves as men or women.

Only participants with complete participation were included in the analysis, meaning donating a blood sample and filling out the questionnaire. Participants with PCR confirmed SARS-CoV-2 infection (*n* = 286) were excluded (seroprevalence of PCR confirmed participants: 80.1% (95% CI 75.4%–84.7%)). Only seropositive participants unaware of their past SARS-CoV-2 infection (untested) and seronegative participants were included in the analysis (Fig. 1). Seronegative participants served as a reference group, providing an overview of background symptoms – symptoms experienced in the general population caused by other common cold or flu-like viruses – enabling correction for these background symptoms by comparing the proportion of experienced symptoms between seronegative and seropositive adults. The study population was comparable to the source population (all adults of the province of Limburg, the Netherlands) in the distribution of residence over the provincial municipalities and in age distribution.

### Data collection

Data was collected between 28 October and 23 December 2020 and consisted of donating a blood sample and completing an online questionnaire.

Online participation registration was possible via market research software from Crowdtech (ISO-20252 and ISO-27001 certified, London, UK). Registration was followed by an appointment for blood drawing, where one 10 ml EDTA tube was taken by venepuncture. Blood samples were collected by trained and certified health professionals and analysed at the Medical Microbiology Laboratory of the Maastricht University Medical Centre+ (MUMC+). Presence of Ig total SARS-CoV-2 antibodies was determined in serum samples of all participants using the Wantai SARS-CoV-2 Ab ELISA test (Beijing Wantai Biological Pharmacy Enterprise Co., Ltd., Beijing, China) in accordance with the manufacturer's instructions [13]. Result of the antibody test was a dichotomous outcome measure (positive/negative). Borderline outcomes were considered positive.

### Questionnaire

The questionnaire covered general demographics (sex, age, level of education), occupation (working in healthcare, catering or other sectors), underlying diseases (absence of spleen, diabetes, immune disorder, liver disease, lung disease, kidney disease, cancer, organ/bone marrow transplant, rheumatoid arthritis, obesity (body mass index > 30), cardiovascular disease, skin disorder, neurological disorder, gastrointestinal disorder and depression), having hay fever (yes/no), and experienced (COVID-19 related) symptoms.

### Variables used in analysis

Age categories were created (18–60 years and 60 years and older), based on previous studies investigating age differences in experienced COVID-19 symptoms [14]. Level of education was dichotomised into practically trained (no, lower general, lower vocational, general secondary and secondary vocational education) and theoretically trained (higher general, pre-university, higher professional and scientific education).

Symptoms were asked over the periods February-March (after the first COVID-19 case was diagnosed in The Netherlands), April–June, and May–November 2020 (before serological testing in the current study).

A list of 23 symptoms was composed using the expertise of infectious diseases physicians and national and international literature. The list included elevated body temperature (37.5–38.0 °C), fever (>38 °C), cold shivers, general malaise, coughing, sore throat, runny nose, shortness of breath, pain when breathing, fatigue, myalgia, headache, stomach ache, diarrhoea, nausea, vomiting, loss of smell, loss of taste, loss of appetite, dizziness, irritability, eye pain and skin abnormalities. The number of days each symptom lasted in the specified period was counted.

Experiencing a symptom was categorised in two categories for each symptom; did not experience a specific symptom during all three periods or experienced a specific symptom at least once during one of the periods. No order in experiencing symptoms during the three periods was maintained.

### Statistical analysis

No missing data had to be handled, since all questions in the questionnaire were mandatory.

Seroprevalence with 95% confidence intervals (CI) was calculated. Interactions between sex and experienced symptoms were tested, resulting in statistically significant interactions (i.e. effect modification by sex) for elevated body temperature (37.5–38.0 °C), fever (>38.0 °C), cold shivers, general malaise, shortness of breath, headache and loss of appetite. Additional interactions between age groups (18–60 years, 60 years and older) and experienced symptoms were tested, revealing significant interactions for elevated body temperature, fever, cold shivers, general malaise, coughing, sore throat, fatigue, myalgia, headache, stomach ache, diarrhoea, nausea, loss of appetite, dizziness and loss of smell and taste. These significant interactions indicate that the difference in the proportion of experienced symptoms between seropositive and seronegative participants, additionally differs by sex and age subgroups, and thereby underline the importance of stratifying data by sex and age. Analysis was performed using Statistical Package for the Social Sciences (SPSS) version 26.0. A *P*-value <0.05 was valued as statistically significant.

No statistical tests were used to evaluate whether the proportion of experienced symptoms differed by serostatus. Due to the large study population, small differences without any clinical interest might result in statistical significant tests [15]. Therefore, results were focussed on describing proportions of experienced symptoms for different subgroups and differences in proportions were established by visual inspection (eyeballing). To assess possible group differences between participants having hay fever or not, proportions of experienced symptoms were additionally described when participants reporting having hay fever were excluded.

## Results

### Study population and characteristics

A total of 9715 participants completed participation (Table 1). More than half of the participants were women (58.1%) and theoretically trained (52.7%), and on average 49 years old (s.d. = 15 range 18–90 years). Most prevalent reported underlying diseases were obesity (16.1%), and cardiovascular disease (15.1%). Having hay fever was reported by 25.7% of the participants.

**Table 1.** Participant characteristics for total population and stratified according to serostatus

	Total (*n* = 9715)				Seronegative (*n* = 7996)		Seropositive (*n* = 1719)	
Sex	*n*	%	SP (%)	95% CI	*n*	%	*n*	%
Men	4068	41.9	17.3	16.1–18.4	3365	42.1	703	40.9
Women	5643	58.1	18.0	17.0–19.0	4629	57.9	1014	59.0
Other	4	0	–	–	2	0	2	0.1
Age (mean (s.d.))	49 (15)				49 (15)		49 (15)	
Age								
18–60 years	6825	70.3	17.3	12.9–17.3	5644	70.6	1181	68.7
≥60 years	2980	30.7	18.6	13.5–22.5	2352	29.4	538	31.3
Level of education								
Practically	4596	47.3	18.3	17.2–19.4	3755	47.0	841	48.9
Theoretically	5119	52.7	17.2	16.1–18.2	4241	53.0	878	51.1
Occupation								
Healthcare	1477	15.2	22.0	19.9–24.1	1152	14.4	325	18.9
Catering	265	2.7	22.6	17.6–27.7	205	2.6	60	3.5
Other	7973	82.1	16.7	15.9–17.6	6639	83.0	1334	77.6

SP, seroprevalence; s.d., standard deviation.

Women were on average younger (48 years *vs*. 51 years in men), more often practically trained (50.1% *vs*. 43.4% in men), and more frequently working in health care or catering (23.8% *vs*. 9.9% in men), compared to men. Diabetes (5.5% in men *vs*. 2.2% in women) and cardiovascular diseases (18.6% in men *vs*. 12.5% in women) were more prevalent in men, while having hay fever was more prevalent in women (27.3% *vs*. 23.6% in men).

### Seroprevalence

The overall seroprevalence was 17.7% (95% CI 16.9%–18.5%), and comparable between men (17.3% (95% CI 16.1–18.5)) and women (18.0% (95% CI 17.0–19.0)).

### Sex and age differences in experienced symptoms

Independent of age and sex, the proportion of experiencing elevated body temperature, fever, cold shivers, general malaise, fatigue, myalgia, loss of appetite, loss of taste and loss of smell was different by serostatus (Table 2).

**Table 2.** Proportion of experienced symptoms stratified according to serostatus, sex and age

	Men (*n* = 4068)			Women (*n* = 5643)		
	Seronegative (*n* = 3365)	Seropositive (*n* = 703)	Total	Seronegative (*n* = 4629)	Seropositive (*n* = 1014)	Total
Elevated body temperature						
18–59 years	31.9	57.6	36.2	43.1	61.8	46.4
≥60 years	24.1	58.4	30.4	32.9	54.0	36.9
Total	29.2	57.9	34.2	40.5	59.7	43.9
Fever						
18–59 years	23.6	48.3	27.7	28.0	48.0	31.5
≥60 years	14.9	54.6	22.2	23.7	49.6	28.6
Total	20.6	50.6	25.8	26.9	48.4	30.8
Cold shivers						
18–59 years	37.0	51.7	39.4	43.5	54.2	45.4
≥60 years	23.8	44.7	27.6	34.1	48.9	36.9
Total	32.4	49.1	35.3	41.1	52.8	43.2
General malaise						
18–59 years	41.3	60.1	44.5	52.7	69.6	55.7
≥60 years	33.9	67.6	40.1	48.5	73.9	53.3
Total	38.8	62.9	42.9	51.6	70.8	55.1
Coughing						
18–59 years	60.1	64.4	60.8	62.9	65.6	63.4
≥60 years	46.3	56.5	48.1	51.1	59.1	52.6
Total	55.3	61.5	56.4	59.9	63.8	60.6
Sore throat						
18–59 years	59.0	56.7	58.6	69.7	66.5	69.1
≥60 years	39.0	43.5	39.8	48.8	52.9	49.6
Total	52.1	51.8	52.0	64.3	62.8	64.1
Runny nose						
18–59 years	64.4	60.5	63.7	67.1	63.8	66.5
≥60 years	51.2	47.7	50.5	49.9	52.5	50.4
Total	59.8	55.8	59.1	62.7	60.7	62.3
Shortness of breath						
18–59 years	31.0	43.1	33.0	37.3	46.9	39.0
≥60 years	23.7	37.4	26.2	30.4	35.9	31.4
Total	28.4	41.0	30.6	35.6	43.9	37.1
Pain when breathing						
18–59 years	14.0	20.2	15.1	20.6	24.9	21.3
≥60 years	9.4	15.3	10.4	16.9	24.3	18.3
Total	12.4	18.3	13.4	19.6	24.8	20.6
Fatigue						
18–59 years	51.4	69.4	54.4	64.8	80.5	67.6
≥60 years	37.4	69.5	43.3	51.4	75.4	55.9
Total	46.6	69.4	50.5	61.4	79.1	64.5
Myalgia						
18–59 years	40.6	49.4	42.1	44.2	58.3	46.7
≥60 years	28.5	51.1	32.7	35.4	51.4	38.4
Total	36.4	50.1	38.8	41.9	56.4	44.5
Headache						
18–59 years	52.5	63.5	54.3	68.8	73.8	69.7
≥60 years	30.3	49.2	33.8	47.3	60.9	49.8
Total	44.8	58.2	47.1	63.3	70.3	64.6
Stomach ache						
18–59 years	18.3	17.7	18.2	24.8	22.9	24.5
≥60 years	11.0	15.3	11.8	18.3	26.8	19.9
Total	15.8	16.8	16.0	23.2	24.0	23.3
Diarrhoea						
18–59 years	28.2	26.8	28.0	28.4	28.7	28.5
≥60 years	20.3	23.7	21.0	25.8	32.2	27.0
Total	25.5	25.6	25.5	27.8	29.7	28.1
Nausea						
18–59 years	18.2	20.6	18.6	26.9	24.3	26.4
≥60 years	12.3	18.3	13.4	21.4	33.7	23.7
Total	16.2	19.8	16.8	25.5	26.8	25.7
Vomiting						
18–59 years	7.6	8.6	7.8	10.6	8.9	10.3
≥60 years	4.6	3.8	4.5	9.0	9.4	9.1
Total	6.6	6.8	6.6	10.2	9.1	10.0
Loss of appetite						
18–59 years	18.1	39.0	21.6	28.5	50.5	32.4
≥60 years	14.5	46.9	20.5	28.8	59.8	34.7
Total	16.9	42.0	21.2	28.6	53.1	33.0
Dizziness						
18–59 years	20.0	20.2	20.0	27.9	30.6	28.3
≥60 years	16.4	19.8	17.0	23.0	32.6	24.8
Total	18.7	21.1	19.0	26.6	31.2	27.4
Irritability						
18–59 years	13.9	19.5	14.8	16.5	19.4	17.0
≥60 years	11.4	18.3	12.7	11.4	15.2	12.1
Total	13.0	19.1	14.1	15.2	18.2	15.7
Eye pain						
18–59 years	10.4	11.8	10.6	12.2	14.5	12.6
≥60 years	8.4	6.5	8.1	13.8	13.8	13.8
Total	9.7	9.8	9.7	12.6	14.3	12.9
Skin abnormalities						
18–59 years	3.9	4.3	3.9	5.5	8.0	5.9
≥60 years	5.1	5.3	5.1	6.0	5.8	5.9
Total	4.3	4.7	4.4	5.6	7.4	5.9
Loss of taste						
18–59 years	10.2	51.9	17.2	12.1	58.0	20.2
≥60 years	7.0	42.4	13.5	13.9	49.6	20.6
Total	9.1	48.4	15.9	12.6	55.7	20.3
Loss of smell						
18–59 years	9.0	51.9	16.2	11.7	56.8	19.6
≥60 years	7.2	38.9	13.0	11.7	47.5	18.5
Total	8.4	47.1	15.1	11.7	54.2	19.3
No symptom experienced						
18–59 years	11.2	3.6	9.9	6.9	2.2	6.1
≥60 years	22.4	6.9	19.6	17.9	4.0	15.3
Total	15.1	4.8	13.3	9.8	2.7	8.5

Men aged 18–60 years revealed larger differences in the proportion of experienced symptoms by serostatus for all symptoms, compared to women in the same age category, except for myalgia, loss of appetite, dizziness, loss of taste and loss of smell. The proportion of experiencing pain when breathing, headache and irritability differed more by serostatus in men, compared to women aged 18–60 years. Stomach ache, nausea and dizziness did not differ by serostatus in both sexes in the age of 18–60 years.

In men aged 60 years and older, larger differences in the proportion of experienced symptoms by serostatus were established for most symptoms compared to women, except for pain when breathing, stomach ache, nausea, dizziness, loss of taste and loss of smell. Stomach ache, nausea and dizziness differed more by serostatus in women aged 60 years and older compared to men, while the proportion of irritability differed more by serostatus in men aged 60 years and older only compared to women. The difference in the proportion of experiencing pain when breathing by serostatus was comparable between men and women aged 60 years and older (Table 2).

Furthermore, no differences by serostatus were observed for the prevalence of coughing, sore throat, runny nose, diarrhoea, vomiting, eye pain and skin abnormalities, independent of sex and age (Table 2).

The proportion of participants who did not experience any symptoms was higher in men (18–60 = 9.9% and ≥60 = 19.6%) than women (18–60 = 6.1% and ≥60 = 15.3%), independent of serostatus. However, this difference was minimal between seropositive men (18–60 = 3.6% and ≥60 = 6.9%) and seropositive women (18–60 = 2.2% and ≥60 = 4.0%) (Table 2).

Minimal differences in experienced symptoms by serostatus (0.1%–3.9%) were observed when participants having hay fever were excluded (Supplementary Table S1).

## Discussion

This strongly designed study presents distinct sex and age differences in experienced symptoms in a large cohort of individuals who were previously unaware of their SARS-CoV-2 infection and where the previous infection was established by serology. The study was conducted in the first 8 months of the pandemic before the introduction of COVID-19 vaccinations and during the Wuhan strain of SARS-CoV-2. The majority of the symptoms was more frequently experienced in men compared to women. The proportion of experiencing pain when breathing, headache and irritability was different by serostatus in men, but not in women aged 18–60 years. Stomach ache, nausea, and dizziness did not differ by serostatus in both sexes in the age of 18–60 years. Stomach ache, nausea and dizziness only differed by serostatus in women aged 60 years and older, while the proportion of irritability differ by serostatus in men aged 60 years and older only. Proportion of experiencing pain when breathing was not different by serostatus in both men and women aged 60 years and older. Displaying differences in experienced symptoms for age and sex subgroups increases the opportunity of early infection detection, especially as COVID-19 manifests with many symptoms which are also common in other viral infections.

This study is the first among (untested) unaware COVID-19 cases, including a negative reference group, stratifying for sex and age, highlighting the novelty of our study. Previous studies among confirmed COVID-19 cases counted cough, chills, fever and shortness of breath more often in men [2–4], while nausea, headache and loss of smell were more common in women [4, 5], independent of age. In contrast, we only observed a difference in the proportion of experiencing nausea and headache by serostatus in women aged 60 years and older. We used a population which also included seronegative participants, while previous studies only included (severely ill) confirmed COVID-19 cases, without using a negative control or reference group. Including a reference group facilitates correction for background symptoms, not caused by SARS-CoV-2 infection. Nausea and headache can be suggested to be background symptoms often experienced in younger women, as we showed a higher proportion of experiencing nausea (18–60 = 26.9% and ≥60 = 21.4%) and headache (18–60 = 68.8% and ≥60 = 47.3%) in women aged 18–60 years. Previous research showed nausea to be more frequently experienced in female than male university students [16]. Also headache was more prevalent in women and steadily decreased with older age [17]. The comparable proportion of experiencing nausea and headache by serostatus in women aged 18–60 years might therefore be explained by correcting for the general occurrence of nausea in seronegative women, which was not done in previous research.

Severity of disease and SARS-CoV-2 antibody responses can be addressed as possible explanations for the greater differences in proportions of experienced symptoms by serostatus in men. Several European countries show that men are prone to have a more severe COVID-19 course compared to women [18], accompanied by a steadily elevated men-to-women case fatality rate through all age groups [19]. A better-sustained antibody response is observed when significant symptoms are experienced during infection [11]. The participants in our study were untested for COVID-19, making them in all probability unaware of the time of infection, subsequently excluding the possibility to determine severity of their acute infection. The overall course of their infection was probably mild, as severe illness would have required hospitalisation accompanied with COVID testing. Nevertheless, within this mild course of infection severity can differ. Unfortunately, the severity of the experienced symptoms was not asked in our study. Additionally, the male sex is associated with higher anti-SARS-CoV-2 antibody production in convalescent patients [20] and higher pro-inflammatory cytokine expression during SARS-CoV-2 infection [21], although antibody responses are in general higher in women, resulting from greater humoral and cell-mediated immune responses to infection [20]. In our study, serostatus was a dichotomous outcome variable, which does not indicate the level of the antibody response (antibody titre). Therefore, it is unclear whether the greater experiencing of symptoms in men is due to immune-related sex differences or a more severe course of infection men are prone to.

Furthermore, co-morbidities that are more prevalent in men, including chronic lung disease, hypertension, cardiovascular disease and diabetes, have been associated with COVID-19 severity [18, 22, 23], and thus might explain the larger difference in the proportion of experienced symptoms by serostatus in men. The prevalence of diabetes and cardiovascular disease was higher in men compared to women in our study as well. Despite, current literature points out that several diseases can develop after a SARS-CoV-2 infection, for example metabolic disorders and respiratory and cardiovascular conditions [24]. Due to the cross-sectional study design, we cannot study the possible role of underlying diseases and experience of symptoms in different sex and age subgroups, however it might contribute.

Additionally, hormonal differences might explain different outcomes following infection. Oestrogens might play a role in reducing the severity of disease [25]. As oestrogen levels are specifically higher in women of the reproductive ages, sex differences in symptom experiencing would be expected to be greater in this age category. Our results are inconclusive, as we observed sex differences in the proportion of headache and pain when breathing in 18–60 years olds, which were not present in 60 year olds, and, on the other hand, we showed sex differences in 60 years and older participants, for example for stomach ache and dizziness.

Some strengths and limitations of our study need to be discussed. We conducted a large, community-based study in which all of the seropositive participants were unaware of their past SARS-CoV-2 infection, which diminishes the possibility of information bias that is differential by COVID-status, and increasing the generalisability of our results. Furthermore, we were able to include possible asymptomatic participants and participants experiencing only mild symptoms, a population often not included in research focused on COVID-19 symptoms. In relation to this, a large proportion of the population tested seronegative, providing a sufficient reference group experiencing background symptoms.

The first limitation is the possibility of recruitment bias, using the convenience sampling method. Nevertheless, the study population was comparable to the source population in the distribution of residence over the provincial municipalities and in age distribution. Unfortunately, comparing occupation or socioeconomic level between the study and source population was not feasible, as required data on these factors were lacking. Furthermore, selection bias could have occurred as registration for participation and filling out the questionnaire was done digitally. To minimise this, a telephone helpline was made available for assistance. Regarding the external generalisability of our results, we acknowledge that only including COVID-19 cases unaware of their infection, probably accompanied by a relatively mild course of disease, constrains the possibility to generalise our results to (hospitalised) COVID-19 cases with severe illness. Yet, our study specifically aimed to include participants unaware of their previous infection and results are only stated based on this population. In addition, symptoms were reported over a longer time period, possibly subjected to recall bias. However, we expect that this bias is non-differential, as the general population is expected to be more aware about their experienced health or might be more triggered to report symptoms compared to the time before COVID-19, possibly leading to over-reporting of symptoms. Since the start of the pandemic, being aware of changes in health status and monitoring symptoms were emphasised and extensively communicated by the Dutch government and public health authorities. Especially during the first months of the pandemic when testing possibilities were limited. The increased concern about general health during the pandemic is supported by a significant increase in the experience of anxiety and panic/somatic symptoms observed when comparing assessments of December 2014 and May 2020 in a sample of adolescents [26]. It can be assumed that this increased awareness is comparable between seropositive and seronegative participants, as seropositive participants were untested and unaware of their infection. Third, we were unable to establish whether symptoms were experienced simultaneously. This eliminated the possibility to assess clusters or combinations of symptoms frequently experienced. Because none of the seropositive participants had a confirmed infection, the possible time of infection was not known, excluding the possibility to follow up the persistence of symptoms after infection. Therefore, we probably measured a mixture of acute and long-term (long COVID) experienced symptoms after SARS-CoV-2 infection, which we were not able to distinguish.

To conclude, we assessed differences in the proportion of a great diversity of experienced symptoms and serostatus in a large cohort of both unaware (untested) seropositive compared with seronegative reference participants. The observed great variety of experienced symptoms challenges the possibility to define a testing policy based on relevant COVID-19-related symptoms. We showed sex and age differences in experienced symptoms, mainly with men demonstrating a higher proportion of experiencing certain symptoms than women. This implies that there might be an added value for defining sex and age-specific COVID-19-related symptom profiles for testing strategies and infection prevention policies. As governments currently move away from mass testing, the importance of symptom monitoring by the general population is emphasised again, like it was in the first months of the pandemic when testing was restricted. However, newly emerging variants and meanwhile widely administered vaccines continue to be a challenge, as they may alter symptomatology and thereby our understanding of COVID-19. Future research and policy makers should take possible sex and age differences and its importance with respect to acute and long-term COVID-19 outcomes into account.

## Data Availability

Data cannot be shared publicly because the data contains potentially identifying patient information. Data are available on request from the head of the data-archiving South Limburg Public Health Service (contact via Helen.Sijstermans@ggdzl.nl) for researchers who meet the criteria for access to confidential data.

## Supplementary material

For supplementary material accompanying this paper visit http://doi.org/10.1017/S0950268822001339. click here to view supplementary material
