# Supplementary material for: Sex and age differences in the proportion of experienced symptoms by SARS-CoV-2 serostatus in a community-based cross-sectional study
Source: Epidemiol Infect. 2022 Aug 10;150:e157. doi: 10.1017/S0950268822001339 (PMC9472034; doi:10.1017/S0950268822001339)
Supplement: Supplementary file 1 [file S0950268822001339sup001.docx]

Epidemiology and Infection, Sex and age differences in the proportion of experienced symptoms by SARS-CoV-2 serostatus in a community-based cross-sectional study, Demi ME Pagen^1,2*^, Stephanie Brinkhues^1,2^, Nicole HTM Dukers-Muijrers^1,3^, Casper DJ den Heijer^1,2^, Noortje Bouwmeester-Vincken^4^, Daniëlle AT Hanssen^5^, Linda M van de Laar^1^, Inge HM van Loo^5^, Paul HM Savelkoul^5,6^, Christian JPA Hoebe^1,2,5^, Supplementary Material

Supplementary Table S1. Proportion of experienced symptoms stratified according to serostatus, sex and age, excluding participants having hay fever

|  | Men (n=3107) | | | Women (n=4105) | | |
| --- | --- | --- | --- | --- | --- | --- |
|  | Seronegative  (n=2576) | Seropositive  (n=531) | Total | Seronegative  (n=3318) | Seropositive  (n=787) | Total |
| Elevated body temperature | |  |  |  |  |  |
| 18-59 years | 31.3 | 56.6 | 35.4 | 41.1 | 62.0 | 45.1 |
| ≥60 years | 24.5 | 56.4 | 30.3 | 34.2 | 53.0 | 37.9 |
| Total | 28.7 | 56.5 | 33.4 | 39.1 | 59.3 | 43.0 |
| Fever |  |  |  |  |  |  |
| 18-59 years | 23.6 | 48.9 | 27.7 | 26.5 | 48.8 | 30.7 |
| ≥60 years | 15.2 | 54.1 | 22.3 | 23.6 | 49.1 | 28.6 |
| Total | 20.4 | 51.0 | 25.6 | 25.6 | 48.9 | 30.1 |
| Cold shivers |  |  |  |  |  |  |
| 18-59 years | 36.5 | 51.1 | 38.9 | 42.2 | 54.4 | 44.5 |
| ≥60 years | 23.5 | 45.9 | 27.6 | 32.5 | 50.0 | 35.9 |
| Total | 31.5 | 49.0 | 34.5 | 39.4 | 53.1 | 42.0 |
| General malaise |  |  |  |  |  |  |
| 18-59 years | 40.6 | 58.8 | 43.6 | 50.1 | 70.5 | 54.0 |
| ≥60 years | 32.6 | 65.9 | 38.6 | 48.0 | 72.4 | 52.7 |
| Total | 37.5 | 61.8 | 41.7 | 49.5 | 71.0 | 53.6 |
| Coughing |  |  |  |  |  |  |
| 18-59 years | 58.9 | 64.3 | 59.8 | 61.5 | 65.2 | 62.2 |
| ≥60 years | 44.4 | 58.2 | 46.9 | 50.2 | 58.6 | 51.8 |
| Total | 53.3 | 61.8 | 54.7 | 58.2 | 63.3 | 59.2 |
| Sore throat |  |  |  |  |  |  |
| 18-59 years | 57.6 | 55.6 | 57.3 | 68.3 | 66.5 | 67.9 |
| ≥60 years | 37.7 | 42.7 | 38.6 | 47.6 | 52.2 | 48.5 |
| Total | 50.0 | 50.3 | 50.0 | 62.4 | 62.3 | 62.3 |
| Runny nose |  |  |  |  |  |  |
| 18-59 years | 63.4 | 59.8 | 62.8 | 65.6 | 62.0 | 64.9 |
| ≥60 years | 50.5 | 49.1 | 50.2 | 48.5 | 50.0 | 48.8 |
| Total | 58.4 | 55.4 | 57.9 | 60.7 | 58.4 | 60.3 |
| Shortness of breath | |  |  |  |  |  |
| 18-59 years | 28.1 | 41.5 | 30.3 | 34.0 | 47.4 | 36.5 |
| ≥60 years | 22.0 | 38.2 | 24.9 | 29.3 | 34.9 | 30.4 |
| Total | 25.8 | 40.1 | 28.2 | 32.6 | 43.7 | 34.8 |
| Pain when breathing | |  |  |  |  |  |
| 18-59 years | 13.0 | 19.0 | 14.0 | 19.0 | 25.9 | 20.3 |
| ≥60 years | 9.0 | 16.4 | 10.3 | 16.9 | 23.7 | 18.2 |
| Total | 11.5 | 17.9 | 12.6 | 18.4 | 25.3 | 19.7 |
| Fatigue |  |  |  |  |  |  |
| 18-59 years | 50.6 | 68.5 | 53.5 | 63.5 | 80.5 | 66.7 |
| ≥60 years | 35.2 | 67.7 | 41.1 | 49.3 | 75.0 | 54.3 |
| Total | 44.7 | 68.2 | 48.7 | 59.4 | 78.9 | 63.2 |
| Myalgia |  |  |  |  |  |  |
| 18-59 years | 40.3 | 49.2 | 41.8 | 42.7 | 59.6 | 46.0 |
| ≥60 years | 27.2 | 50.0 | 31.4 | 33.2 | 49.1 | 36.3 |
| Total | 35.3 | 49.5 | 37.7 | 40.0 | 56.5 | 43.2 |
| Headache |  |  |  |  |  |  |
| 18-59 years | 50.5 | 62.4 | 52.5 | 66.6 | 75.3 | 68.3 |
| ≥60 years | 28.5 | 47.7 | 32.0 | 44.7 | 59.9 | 47.7 |
| Total | 42.0 | 56.3 | 44.5 | 60.3 | 70.8 | 62.3 |
| Stomach ache |  |  |  |  |  |  |
| 18-59 years | 17.5 | 16.7 | 17.4 | 22.7 | 22.9 | 22.7 |
| ≥60 years | 10.1 | 15.0 | 11.0 | 17.3 | 29.7 | 19.7 |
| Total | 14.6 | 16.0 | 14.9 | 21.1 | 24.9 | 21.9 |
| Diarrhoea |  |  |  |  |  |  |
| 18-59 years | 27.4 | 25.4 | 27.1 | 27.1 | 27.7 | 27.3 |
| ≥60 years | 19.4 | 23.2 | 20.1 | 23.8 | 31.9 | 25.4 |
| Total | 24.3 | 24.5 | 24.4 | 26.2 | 29.0 | 26.7 |
| Nausea |  |  |  |  |  |  |
| 18-59 years | 17.9 | 19.0 | 18.0 | 25.2 | 24.9 | 25.1 |
| ≥60 years | 11.6 | 18.2 | 12.8 | 18.9 | 34.1 | 21.9 |
| Total | 15.5 | 18.6 | 16.0 | 23.4 | 27.6 | 24.2 |
| Vomiting |  |  |  |  |  |  |
| 18-59 years | 7.7 | 6.8 | 7.5 | 10.6 | 9.4 | 10.4 |
| ≥60 years | 4.6 | 4.5 | 4.6 | 8.1 | 9.9 | 8.4 |
| Total | 6.5 | 5.8 | 6.4 | 9.9 | 9.5 | 9.8 |
| Loss of appetite |  |  |  |  |  |  |
| 18-59 years | 18.4 | 38.6 | 21.7 | 26.7 | 52.3 | 31.6 |
| ≥60 years | 13.4 | 49.1 | 19.9 | 28.3 | 59.9 | 34.5 |
| Total | 16.5 | 42.9 | 21.0 | 27.2 | 54.5 | 32.4 |
| Dizziness |  |  |  |  |  |  |
| 18-59 years | 19.7 | 18.6 | 19.6 | 26.4 | 30.5 | 27.2 |
| ≥60 years | 15.3 | 20.9 | 16.4 | 22.9 | 33.2 | 24.9 |
| Total | 18.1 | 19.6 | 18.3 | 25.4 | 31.3 | 26.5 |
| Irritability |  |  |  |  |  |  |
| 18-59 years | 12.5 | 19.6 | 13.7 | 15.3 | 19.5 | 16.1 |
| ≥60 years | 10.7 | 18.2 | 12.1 | 11.1 | 15.5 | 12.0 |
| Total | 11.8 | 19.0 | 13.0 | 14.1 | 18.3 | 14.9 |
| Eye pain |  |  |  |  |  |  |
| 18-59 years | 10.3 | 10.0 | 10.3 | 11.0 | 15.1 | 11.8 |
| ≥60 years | 8.1 | 6.4 | 7.8 | 12.7 | 12.5 | 12.7 |
| Total | 9.5 | 8.5 | 9.3 | 11.5 | 14.4 | 12.0 |
| Skin abnormalities | |  |  |  |  |  |
| 18-59 years | 4.1 | 3.9 | 4.1 | 4.8 | 6.8 | 5.2 |
| ≥60 years | 4.7 | 5.0 | 4.8 | 5.5 | 6.5 | 5.7 |
| Total | 4.3 | 4.3 | 4.3 | 5.0 | 6.7 | 5.3 |
| Loss of taste |  |  |  |  |  |  |
| 18-59 years | 10.1 | 50.2 | 16.7 | 11.8 | 59.3 | 20.8 |
| ≥60 years | 6.7 | 44.1 | 13.5 | 14.4 | 49.1 | 21.2 |
| Total | 8.8 | 47.6 | 15.4 | 12.5 | 56.3 | 20.9 |
| Loss of smell |  |  |  |  |  |  |
| 18-59 years | 9.1 | 50.8 | 15.9 | 11.1 | 57.5 | 19.9 |
| ≥60 years | 6.8 | 41.8 | 13.1 | 12.1 | 48.3 | 19.2 |
| Total | 8.2 | 47.1 | 14.8 | 11.4 | 54.8 | 19.7 |
| No symptom experienced | |  |  |  |  |  |
| 18-59 years | 12.4 | 3.9 | 11.0 | 7.6 | 2.0 | 6.5 |
| ≥60 years | 23.4 | 6.8 | 20.4 | 19.5 | 4.7 | 16.6 |
| Total | 16.7 | 5.1 | 14.7 | 11.0 | 2.8 | 9.4 |
|  | | | | | | |
